# Interrupting Neuron—Tumor Interactions to Overcome Treatment Resistance

**DOI:** 10.3390/cancers12123741

**Published:** 2020-12-12

**Authors:** Patrick J. Hunt, Katherine E. Kabotyanski, George A. Calin, Tongxin Xie, Jeffrey N. Myers, Moran Amit

**Affiliations:** 1 Medical Scientist Training Program, Baylor College of Medicine, Houston, TX 77030, USA; PJHunt@bcm.edu (P.J.H.); katherine.kabotyanski@bcm.edu (K.E.K.); 2 Department of Neurosurgery, Division of Surgery, The University of Texas MD Anderson Cancer Center, Houston, TX 77030, USA; 3 Jan and Dan Duncan Neurological Research Institute, Texas Children’s Hospital, Houston, TX 77030, USA; 4 Translational Molecular Pathology, Division of Pathology, The University of Texas MD Anderson Cancer Center, Houston, TX 77030, USA; gcalin@mdanderson.org; 5 Department of Head and Neck Surgery, Division of Surgery, MD Anderson Cancer Center, Houston, TX 77030, USA; txxie@mdanderson.org (T.X.); jmyers@mdanderson.org (J.N.M.)

**Keywords:** neurotrophic growth, cancer progression, microRNA, tumor microenvironment

## Abstract

**Simple Summary:**

Solid cancers take advantage of the surrounding tissue to stimulate their own growth, to promote their spread, and to escape anticancer immune responses and treatments. Neurons are an important newly identified target for tumors because they can provide all of these benefits and are found throughout the body. Neurons communicate using chemical signals, many of which can be recognized and leveraged by tumor cells. Tumors, in turn, manipulate neurons by sending local signals that drive the growth of neurons into the body of the tumor. In tandem with local signaling, tumors transmit protein and RNA messengers within extracellular vesicles that travel through the bloodstream and other bodily fluids. This long-range tumor signaling is a growing area of research that allows for new diagnostic and therapeutic approaches. Ongoing clinical trials will uncover methods of disrupting tumor–neuron communication for the benefit of patients.

**Abstract:**

Neurons in the tumor microenvironment release neurotransmitters, neuroligins, chemokines, soluble growth factors, and membrane-bound growth factors that solid tumors leverage to drive their own survival and spread. Tumors express nerve-specific growth factors and microRNAs that support local neurons and guide neuronal growth into tumors. The development of feed-forward relationships between tumors and neurons allows tumors to use the perineural space as a sanctuary from therapy. Tumor denervation slows tumor growth in animal models, demonstrating the innervation dependence of growing tumors. Further in vitro and in vivo experiments have identified many of the secreted signaling molecules (e.g., acetylcholine, nerve growth factor) that are passed between neurons and cancer cells, as well as the major signaling pathways (e.g., MAPK/EGFR) involved in these trophic interactions. The molecules involved in these signaling pathways serve as potential biomarkers of disease. Additionally, new treatment strategies focus on using small molecules, receptor agonists, nerve-specific toxins, and surgical interventions to target tumors, neurons, and immune cells of the tumor microenvironment, thereby severing the interactions between tumors and surrounding neurons. This article discusses the mechanisms underlying the trophic relationships formed between neurons and tumors and explores the emerging therapies stemming from this work.

## 1. Introduction

Neurodevelopment is an early step in embryogenesis that provides a framework for further development throughout human life. It is not surprising, then, that shared mechanisms of growth, maintenance, and repair are implicated in the proliferation of tumors. The nervous system is a highly complex network of nerve fibers that extends throughout the human body and plays a critical role in sensing, communicating with, and responding to both our external and internal environments. In vertebrates, the nervous system is divided into the central nervous system, which includes the brain and spinal cord, and the peripheral nervous system, which includes the nerves and ganglia that connect the central nervous system to the rest of the body. Nervous tissue is composed of neurons, which transmit chemical and electrical signals, as well as a variety of specialized cells called neuroglia that provide structural support, electrical insulation, nutrients (including glucose, lipids, and ions) [1], and growth signals (thrombospondins, steroids, etc.) to neurons [2,3].

The differentiation of neurons and glia from pluripotent stem cell precursors occurs early in embryogenesis [4]. Guided by chemical signals, immature neurons migrate and branch to establish their final architecture [5,6]. Although this predominantly occurs during embryonic development, growing evidence [7,8,9,10,11] demonstrates that neurogenesis continues in specific neurogenic niches throughout life. The growth and survival of neurons is subsequently regulated by neurotransmitters and neurotrophins, which include nerve growth factor (NGF) and brain-derived neurotrophic factor (BDNF). These signals are released endogenously by nerve cells and by the target tissues that they ultimately innervate [12,13]. The nervous system overlaps in location and function with the body’s circulatory system [14]. Together, these closely related systems are essential to the proper development, homeostasis, repair, and regeneration of healthy tissues [15,16,17].

Solid tumors have also evolved mechanisms to capitalize on these nutrient-rich neurovascular microenvironments [18]. The requirements for substantial growth and lasting survival in cancer, as in development, make neural signaling a prime target for exploitation by malignant tissues. The appropriation of neurotransmitters and neurotrophins is therefore a recurrent theme in many tumors, including cancers of the brain, skin, prostate, pancreas, and stomach [19]. It is now clear that nerve cells are not passive bystanders sustaining tumor growth but actually help orchestrate tumor initiation and metastatic progression [20]. 

In this review, we discuss how neuron–tumor interactions serve mutually beneficial functions and allow cancerous cells to proliferate while remaining undetected by the body’s defense systems. We also discuss how the molecules involved in these interactions can serve as important biomarkers for identifying the disease state and categorizing disease progression. Finally, we examine how these molecular biomarkers can be used to develop new therapeutic methods and measure treatment responses.

## 2. Neuron—Tumor Communication Drives Tumor Growth

Significant progress has been made in recent years towards understanding the mechanisms by which nerves and tumors communicate with one another. The interplay between peripheral nerves and the tumor microenvironment (TME) is a symbiotic dance that takes advantage of the body’s existing developmental infrastructure while simultaneously circumventing physiologic mechanisms of detection. One of the earliest discoveries of neuron–tumor interaction was a report of the presence of tumor cells within and surrounding peripheral nerves. First described in head and neck cancers in the 19th century, this process of perineural invasion (PNI) has now been identified as a pathologic feature associated with high recurrence rates and poor prognosis in head and neck, pancreatic, colorectal, liver, stomach, prostate, and cervical cancers [21,22]. Electron microscopy studies of perineural structures have shown that peripheral nerve sheaths are not easily penetrable and that PNI does not rely on lymphatic or vascular invasion, existing instead as an independent well-orchestrated process [23,24]. Once shown to be a unidirectional cancer-induced event, further research has demonstrated reciprocal molecular interactions between the TME and the specific peripheral nerve microenvironment, called the perineural niche [25]. The release of neurotransmitters—such as catecholamines and acetylcholine—by nerves into the vicinity of cancer and stromal cells, along with the secretion of neurotrophic growth factors such as NGF and BDNF, and miRNAs by cancer cells, drives the crosstalk that ultimately enables tumor progression. To maximize their access to these neuronally released signals, cancer cells form tripartite synapses with the pre-synaptic and post-synaptic terminals of nearby neurons [26]. These tumor cells concomitantly express neurotransmitter receptors, including NMDA receptors, which, when activated, drive neuron–tumor trophic interactions [27,28,29].

Several elements, including extracellular matrix components, immune circuitry, and the cell signaling machinery, are involved in neuron–tumor interactions. In vitro models of PNI in dorsal root ganglia co-cultured with prostate cancer cells have shown that enhanced neurite outgrowth toward cancer colonies accompanies cancer migration toward the central ganglion, indicating a bidirectional growth advantage [30]. The addition of a mixture of human prostate stromal cells to this model further enhanced growth for both nerves and the prostate cancer cells, indicating that support cells and paracrine signaling mechanisms are also involved in these trophic relationships [31]. Recruitment of inflammatory cells in response to PNI further promotes tumor cell migration. Tumor-activated endoneurial macrophages secrete higher levels of glial-derived neurotrophic factor (GDNF). GDNF induces the phosphorylation of RET tyrosine kinase receptors and activates additional kinases in pancreatic ductal adenocarcinoma (PDAC) cells to drive PNI [32]. Other inflammatory mediators, including cytokines and chemokines, are secreted by the nerves in response to injury and help maintain immune cell recruitment and promote regeneration of the invaded nerve. Chemokine receptors, such as CXCR4 and CXCR5, are overexpressed in prostate and colorectal cancers and are associated with increased PNI [33,34]. Knockout of the chemokine ligand 2 (CCL2) receptor (CCR2) in mice significantly diminished PNI of prostate and PDAC cells compared with wild-type controls, revealing that CCL2–CCR2 signaling is a key mechanism of tumor–nerve communication [35]. Together, these findings demonstrate the active roles that nearby cells within the TME play in mediating and supporting neuron–tumor trophic relationships.

Like chemokines, neurotransmitters and neuropeptides also activate G-protein-coupled receptors expressed in tumor cells and help regulate tumor cell migration and metastasis [36]. Two important catecholamines, epinephrine and norepinephrine, are released from the adrenal medulla and sympathetic nerves, particularly in response to stress. Stress-induced and pharmacologic activation of beta-adrenergic receptors in an orthotopic mouse model increased primary pancreatic tumor growth, whereas beta blockade reversed cancer progression [37]. Denervation of sympathetic nerves and genetic deletion of β2- and β3-adrenergic receptors prevented malignant transformation in the prostate gland and significantly slowed prostate tumor xenograft development [38]. Additional studies have shown that norepinephrine activates cancer cell migration in ovarian [39], breast [40], and colon [41] cancers. In vitro stimulation of PDAC cells with norepinephrine promoted PNI along dorsal root ganglia through beta-adrenergic receptor-induced activation of the STAT3 signaling pathway in a concentration-dependent manner. Concordantly, neural invasion was decreased in cells exposed to propranolol, a beta-adrenergic receptor antagonist [42]. Importantly, this study demonstrated that blocking STAT3 in vitro inhibited norepinephrine-induced pancreatic cancer cell migration, invasion, and PNI, and that treatment with a STAT3 phosphorylation inhibitor blocked PNI of pancreatic cancer cells in vivo. Additional studies have shown similar pro-tumorigenic alterations of the TME by STAT3 activation and the notable antitumor effects of STAT3 inhibition in a variety of cancers (for a comprehensive review on IL-6/JAK/STAT3 signaling in cancer, see Johnson et al. [43]). These data suggest that STAT3 signaling warrants further investigation as a mediator of the interaction between sympathetic nerves and cancer cells.

Apart from promoting tumor migration, epinephrine and norepinephrine also modulate tumor immunity by targeting beta-adrenergic receptors present on inflammatory immune cells, such as T lymphocytes, B lymphocytes, natural killer cells, and macrophages [44]. In ovarian cancer cells, activation of beta-adrenergic receptors by epinephrine and norepinephrine increased the production of the proinflammatory cytokines interleukin-6 and interleukin-8, which regulate lymphocyte activity, promote angiogenesis, and ultimately promote cancer cell survival and growth [45,46]. In breast cancer, norepinephrine secretion increased recruitment of CD11b(+)F4/80(+) macrophages into primary tumors and induced pro-metastatic gene expression signatures [47]. Finally, beta-adrenergic signaling was also shown to suppress the cytotoxic function of T lymphocytes and natural killer cells [48,49], thereby allowing tumors to avoid recognition and destruction by the immune system. Thus, increased sympathetic signaling is one method by which cancers indirectly promote their own growth, spread, and immune evasion.

Another neurotransmitter, acetylcholine, is released by parasympathetic nerves and binds to nicotinic and muscarinic acetylcholine receptors. Cholinergic signaling promotes tumor invasion and metastasis in mouse models of prostate cancer, the inhibition of which can be achieved by pharmacologic blockade or genetic disruption of the Type 1 cholinergic muscarinic receptor (CHRM1) [38]. Further evidence implicates parasympathetic cholinergic involvement, specifically at later stages in prostate cancer development: CHRM1 knockout mice developed primary tumors of the same size as wild-type controls, but treatment with carbachol, a muscarinic acetylcholine agonist, produced significantly more metastasis in CHRM1-positive mice. Similarly, tissue samples from prostatectomies in men with prostate cancer illustrated that cholinergic fibers were enriched in cancerous, but not normal, prostate tissue, suggesting that parasympathetic nerves may localize specifically to already developed tumors to signal subsequent tumor dissemination [38]. 

Type 3 muscarinic receptors (CHRM3) are expressed widely in the gastrointestinal tract but are specifically overexpressed in colon [50] and gastric [51] cancers. Removal of the gastric branches of the vagus nerve, the primary component of the parasympathetic nervous system, significantly suppressed gastric tumorigenesis via inhibition of the Wnt signaling pathway in a gastrin overexpression (INS-GAS) mouse model of spontaneous gastric cancer and in gastric cancer patients [52]. In an *Apc^min/+^* mouse model of colon cancer, CHRM3-deficient mice showed a 70% reduction in the number of tumors and an 80% reduction in tumor volume. Although less effective than gene ablation, treatment with scopolamine butylbromide, a muscarinic antagonist, also reduced the number of tumors (by 22%) and tumor volume (by 36%) [53]. Additional studies have demonstrated that human colon cancer cells produce and release acetylcholine, suggesting autocrine mediation of tumor proliferation [54,55].

In addition to cancer cell migration towards and invasion into neural structures, peripheral nerves also grow in response to growth-promoting molecules secreted by tumors. As in embryonic development or nerve regeneration, the release of neurotrophic growth factors and axon guidance molecules from the surrounding tissues stimulates neurite outgrowth and nerve fiber attraction. Upregulation of the NGF precursor (proNGF) and an NGF-induced increase in nerve density have been reported in prostate [56] and gastric [57] cancer cells. Increased neurogenesis is also associated with increased tumor aggression and decreased overall survival in breast [58], prostate [59], and colorectal [60] cancers. In PDAC, tumor cells secrete NGF and BDNF, which act on Tropomyosin-related kinase (Trk) receptors to stimulate nerve growth [61]. Conversely, knockdown of NGF or Trk genes, as well as treatment with a Trk receptor inhibitor, inhibits neurite formation and reduces the proliferation and migration of pancreatic cancer cells in vitro [62]. Ovarian cancer cells express significantly higher levels of BDNF compared with normal ovarian epithelial cells, and high BDNF expression and tumor nerve counts are associated with decreased survival in ovarian cancer patients [63]. BDNF and its receptor, TrkB, are also upregulated in breast [64], prostate [65], gastric [66], lung [67], and cervical [68] cancers. 

Neurotrophin-3 (NT-3) is an additional growth factor that supports the growth of new neurites. NT-3 mRNA levels were found to be 6.5 times greater in PDAC tumor samples compared with controls, and NT-3 expression levels were further enriched specifically in the nerves within these PDAC samples compared with other cell types [69]. NT-3 has also been found to be overexpressed in thyroid, lung, pancreatic, prostate, and ovarian cancers [18]. Vascular endothelial growth factor (VEGF) is another growth factor that is important in establishing trophic interactions between neurons and tumors. Though VEGF activity is not completely understood, this protein has a particularly well-defined role in initiating tumor vascularization and lymph development. Along with these well-documented roles of providing growth factors and avenues of dissemination to tumors, VEGF has been shown to directly promote neuronal outgrowth and survival through rearrangements of the neuronal growth cone cytoskeleton and activation of the Arp2/3 complex [70,71]. 

**Figure 1:**
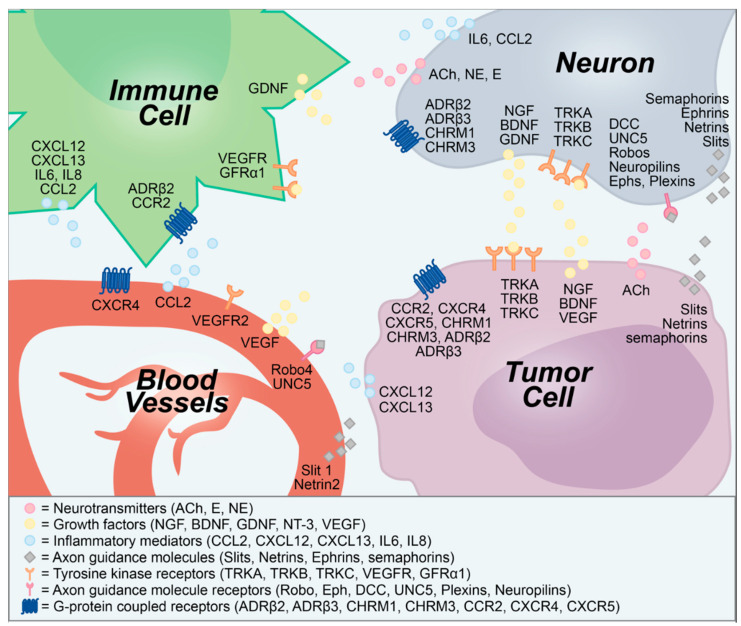
Secreted and membrane-bound molecular signals transmitted between neurons, tumors, blood vessels, and immune cells. ACh, acetylcholine; ADRβ, β-adrenergic receptor; BDNF, brain-derived neurotrophic factor; CCL2, chemokine (C-C motif) ligand 2; CCR2, CCL2 receptor; CHRM, cholinergic muscarinic receptor; CXCL, C-X-C chemokine ligand; CXCR, CXCL receptor; DCC, deleted in colorectal cancer; E, epinephrine; Eph, ephrin receptor; GDNF, glial-derived neurotrophic factor; GFRα1, GDNF family receptor alpha-1; IL, interleukin; NE, norepinephrine; NGF, nerve growth factor; NT-3, neurotrophin-3; TRK, tropomysin-related kinase; UNC5, uncoordinated 5; VEGF, vascular endothelial growth factor.

In addition to growth factors, cancer cells secrete axon guidance molecules, which direct nerve outgrowth into the TME. Slits, netrins, and semaphorins are three families of axon guidance molecules that control neuronal migration and survival in both development and tumor progression. Surprisingly, these molecules also play a role in vascular patterning and tumor angiogenesis [72]. Although angiogenesis and lymphangiogenesis have long been considered key mechanisms in tumor growth, recent evidence suggests that neoneurogenesis may occur as a critical complementary process (Figure 1).

As the mechanisms underlying neurogenic and oncogenic trophic interactions become increasingly well understood, improved methods of sampling tumors, the TME, and peripheral tissue are allowing for the use of trophic signaling molecules as novel diagnostic and prognostic biomarkers of cancer.

## 3. Neurogenic and Oncogenic Trophic Factors as Biomarkers of Disease

**Figure 2:**
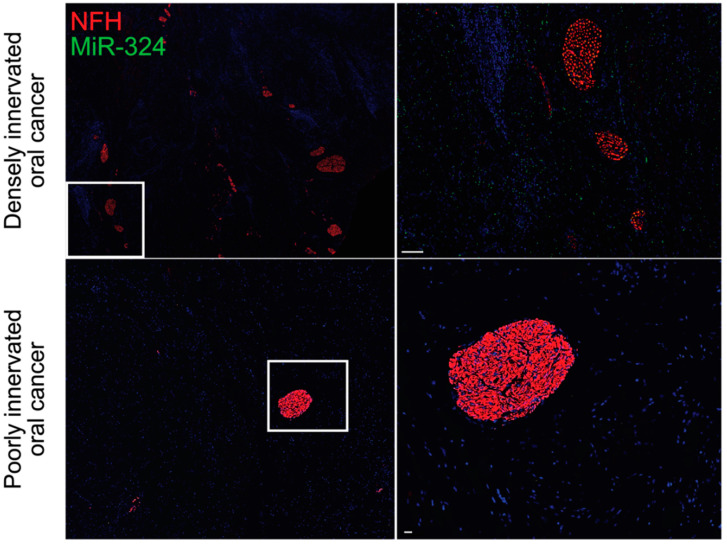
MiR-324 is enriched in densely innervated oral tumors. MiR-324 promotes neuritogenesis within oral tumors, as evidenced by the increased amount of miR-324 in the densely innervated oral tumors (top) when compared with the poorly innervated oral tumors (bottom). Data are original to this manuscript. NFH, neurofilament H. Top scale bar is 100 μm. Bottom scale bar is 30 μm.

There is growing evidence that exosome-mediated communication between tumors and sensory neurons is essential for driving the increased innervation of tumors and the TME. Tumor-derived exosomes carry proteins that regulate the immune system, the TME, and the nearby neural tissue, thereby driving tumor progression and spread [73,74,75,76]. Head and neck squamous cell carcinomas release EphrinB1-laden exosomes, which potentiate nearby sensory neurons for tumor innervation [77]. Similarly, oral cavity squamous cell carcinomas release microRNA (miRNA)-laden exosomes, which drive increased sensory neuron axonogenesis, increased tumor innervation, and a sensory-to-sympathetic switch in these neurons. In these exosomes, miR-34a was found to potently inhibit neuritogenesis in sensory neurons. In contrast, miR-21 and miR-324 within these tumor-released exosomes were found to be strong neuritogenic signals [78]. miR-34a is a candidate biomarker for ovarian cancer and has been shown to correlate with tumor stage and aggression [79]. Additionally, miR-21 has been identified as a biomarker of chemotherapy response in esophageal, lung, pancreatic, and prostate cancers [80,81,82,83]. In these cancers, upregulation of miR-21 promotes treatment resistance by targeting mRNAs that encode important immune-related proteins, including PTEN, PTCD4, and FASL [82,84]. Similar to miR-21, miR-125b drives chemoresistance in breast cancer and non-small cell lung cancer by targeting the mRNA encoding the apoptotic protein BCL2 antagonist killer 1 (BAK1) [85,86]. MiR-324 is a potent neuritogenic signal, which we have found to be profoundly upregulated in densely innervated oral tumors when compared with poorly innervated oral tumors (Figure 2). This increase in intratumoral neurite growth is likely mediated by transcriptional regulation of neuronal differentiation genes, including *Isl1* [78]. 

miRNAs can be isolated and sequenced from body fluids. However, isolating miRNAs from exosomes within body fluids is significantly more sensitive than isolating miRNAs directly from these fluid samples [87]. This is especially important for picking up miRNAs that are not highly expressed and may be difficult to detect. Plasma-derived exosome contents can be used to detect and diagnose different cancers with 95% specificity and 90% sensitivity, thereby serving as a non-invasive and cost-effective liquid biopsy for cancer diagnosis [88]. Exosomes can be rapidly and easily collected from bodily fluids, including peripheral blood, saliva, cerebrospinal fluid, and urine, among others [89,90]. Thus, exosome contents, including miRNAs, mRNAs, small compounds, proteins, and other signals, can serve as biomarkers of disease while also reflecting the messages sent between tumors, neurons, and the TME [91]. 

**Figure 3:**
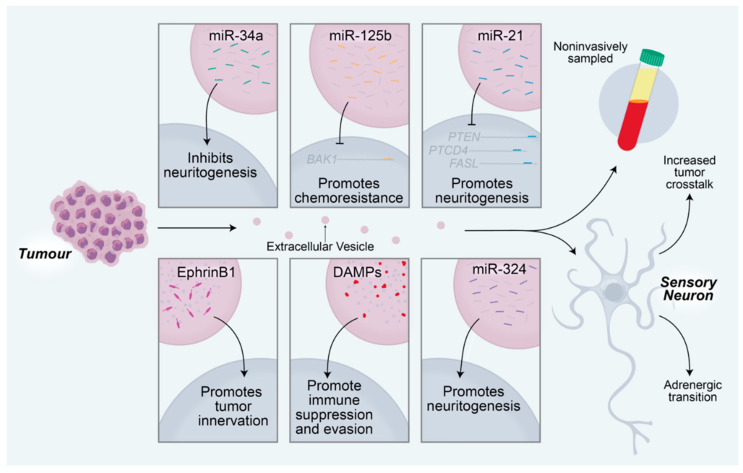
Tumor-derived extracellular vesicles promote tumorigenesis via manipulation of the nearby sensory neurons. MicroRNAs delivered to sensory neurons regulate neuronal changes that subsequently affect tumor activity. MiR-34a from oral cavity squamous cell carcinomas inhibits neuritogenesis in nearby sensory neurons. MiR-125b inhibits BCL2 antagonist killer 1 (BAK1) translation, resulting in chemoresistance of the tumor. MiR-21 promotes sensory neuritogenesis through regulation of PTEN, PTCD4, and FASL transcripts. EphrinB1 proteins delivered to nearby neurons promote the growth of the neurons into the tumor. Damage-associated molecular pattern (DAMP) proteins, including S100A4, S100A13, BSF, and LGALS9, promote immunosuppression, which facilitates immune evasion of the tumor. MiR-324 from OCSCCs promotes neuritogenesis in nearby sensory neurons. Extracellular vesicle contents can be sampled non-invasively from a variety of body fluids, including peripheral blood, saliva, cerebrospinal fluid, and urine, among others. Extracellular vesicles promote increased crosstalk between neurons and tumors and drive a sensory to adrenergic transition in receiving neurons.

Damage-associated molecular pattern (DAMP) proteins are enriched in tumor-derived exosomes. These tumorigenic proteins, which include S100A4, S100A13, BSG, and LGALS9, are immunomodulatory and are associated with immune suppression. Thus, the tumor-driven, exosome-mediated transfer of DAMPs may drive tumor growth and spread by allowing the tumor to evade the anticancer immune response [88]. Exosome sampling will allow for the recognition of these and similar proteins, thereby serving as a potent means of tracking treatment response in cancer patients (Figure 3). This technique is already being adopted to analyze the interplay between the body and different cancers [92,93]. 

New analyses of cancer biopsies that incorporate knowledge gained from studying neuron–tumor interactions are also yielding new diagnostic tools. The protein kinase A (PKA) pathway is an essential mediator of oncogenic stimulation of cancer cells by sympathetic neurons [94]. As a result of these findings, several current clinical trials are testing the efficacy of using PKA levels as a biomarker of metastatic disease in colon cancer (NCT01012804). In one ongoing study, PKA pathway activity is being used as a metric of effect in patients with prostate cancer treated with beta-blockers (NCT03152786). An additional trial is examining the correlation between mu opioid receptor 1 expression and colon cancer progression, using PKA expression as a metric of mu receptor activation (NCT04353882). PKA is an important phosphorylating protein that, when stimulated by cyclic AMP, regulates numerous physiologic processes, including cardiac cell function, neuronal function, and overall cell growth in a variety of healthy tissues. However, this healthy function is dependent on liquid–liquid phase separation of the PKA regulatory subunit within the cell. When this phase separation is disrupted, PKA signaling begins to drive uncontrolled cell growth and spread [95]. Future work will be necessary to uncover how sympathetic stimulation interfaces with this phase-dependent regulation of PKA activity. 

PKA regulates a vast number of downstream substrates and processes. In addition to leveraging PKA activity as a biomarker of disease, proteins involved in PKA function and localization can be used as metrics of disease presence and progression. The regulatory subunits of PKA bind with high affinity to a variety of scaffolding proteins called A kinase anchoring proteins (AKAPs), which localize these PKA subunits to the vicinity of their physiological substrates. These PKA–AKAP interactions have been shown to be important in cancer; anticancer therapies that disrupt these interactions are currently being developed [96,97]. Cyclic AMP activates PKA and is known to be misregulated in ovarian, pancreatic, lung, and hepatocellular cancers. Efforts to measure and manipulate cAMP levels are an additional developing area of therapeutic promise [98]. Further work is needed to determine the role that these interactions play in mediating neuron–tumor relationships.

Downstream of parasympathetic innervation, TP53- and RB1-deficient prostate tumors upregulate SOX2 activity to reprogram cancer cells towards an androgen-insensitive state when exposed to anti-androgen therapy [99]. SOX2 is an essential transcription factor activated by parasympathetic activity during the growth of healthy prostate tissue, and, upon misregulation of this neural-responsive pathway, tumors develop treatment resistance [100]. A current clinical trial is now examining the relationship between SOX2 expression and colon cancer aggression in biopsy samples (NCT01589900). The role of SOX2 in colon cancer treatment resistance will need to be examined in future work.

Interestingly, insulin misregulation has been linked to tumorigenesis and chemoresistance [101,102]. Additionally, insulin signaling acts on the neurons of the peripheral and central nervous systems [103,104]. Whether the observed increase in tumor growth and treatment resistance is mediated by neuron–tumor interactions requires further investigation. 

Examining the molecular relationships among tumors, the TME, and local neurons has uncovered new methods of detecting and diagnosing cancers (Table 1). Additionally, these methods present opportunities to measure treatment response in these tumors and the associated tissues, as well as novel anticancer therapies to slow tumor growth and overcome treatment resistance. 

## 4. Neuron—Tumor Signaling as a Target for Anticancer Therapeutics

The knowledge gained from studying the trophic interactions between nerves and solid tumors is important for the development of novel pharmacologic and surgical therapeutic strategies. Early work in this field physically severed these relationships by surgically denervating tumor beds. Subsequently, a body of work has found that both surgical and pharmacologic denervation of solid tumors results in the slowing or cessation of tumor growth in mice [52,105]. Evidence also demonstrates that increased sympathetic stress is associated with cancer treatment resistance, thereby suggesting that denervation may prevent the development of treatment resistance [106]. Although denervations are more challenging to administer in humans, they have also proven to be effective [107]. However, although effective, denervation is not without symptomatic consequence. Damage to neural plexi during therapeutic denervation of pancreatic or prostate tumors is associated with severe diarrhea, incontinence, and sexual dysfunction. Denervation of tumors in other areas of the body, including the head and neck, is also associated with debilitating side effects [108]. 

For this reason, many have turned to modulating neuronal activity using small molecules. Sympathetic nerves release catecholamines (i.e., norepinephrine, epinephrine), which bind beta adrenergic receptors (e.g., ADRβ2). These bound receptors activate cAMP, which subsequently activates the downstream PKA and exchange protein activated by adenylyl cyclase (EPAC) pathways within the receiving tumor cells, resulting in oncogenic transcriptional changes [94]. Aberrant sympathetic activity also manipulates macrophages, T lymphocytes, and fibroblasts to facilitate tumor growth and spread [109]. In breast, lung, ovarian, and prostate cancers, this beta adrenergic activation can induce a switch from benign to metastatic cancer, resulting in poor clinical outcomes in patients [38,47,109,110]. Consequently, multiple studies have found a clinical benefit to receiving adjuvant beta-adrenergic blockers during treatment of breast, lung, ovarian, and prostate cancers [111,112,113,114,115]. New combinations and uses of beta blockers in prostate and pancreatic tumors are currently being tested (NCT02944201, NCT03838029, NCT04245644, NCT03152786; Table 2). Beta receptor blockade is also effective in re-sensitizing small cell lung cancer to radiation and cisplatin, overcoming treatment resistance by interrupting neuron–cancer trophic interactions [116]. 

Along with these sympathetic blockers, drugs that modulate parasympathetic activity are effective in treating human cancers [100]. However, parasympathetic activity appears to play different roles in cancer progression, depending on the tumor type. Parasympathetic neurons typically release acetylcholine as their main neurotransmitter. Much work has demonstrated the reliance of gastric tumors on parasympathetic cholinergic signaling for growth and spread [52,57]. This cholinergic signaling acts through muscarinic receptors, such as CHRM1, activation of which regulates the mitogen-activated protein kinase (MAPK)/epidermal growth factor receptor (EGFR) and phosphoinositide 3-kinase (PI3K)/AKT pathways. These pathways control aspects of the TME, including CD11b+ myeloid cell activity and TNFα levels [117]. The MAPK/EGFR and PI3K/AKT pathways are also implicated in the development of treatment resistance, mediated by the TME [118,119]. In this way, parasympathetic activity, tumor growth, and treatment resistance are all linked by the same targetable oncogenic pathways. 

In contrast to the cholinergic drive of gastric tumorigenesis, PDAC tumors respond differently to cholinergic stimulation. In these tumors, increased CHRM1 activation suppresses PDAC tumorigenesis [117]—an effect that is currently being tested through the use of muscarinic agonists in patients with PDAC (NCT03572283). Thus, although therapies that target cholinergic signaling have shown promise in mitigating the oncotrophic effects of parasympathetic innervation [120], these treatments must be tailored closely to tumor type. Improvements in single-cell and bulk RNA sequencing assays will allow thorough investigations of cell types and gene expression profiles in tumors [121]. These strategies will become increasingly important because they will allow the targeting of individual genes (i.e., CHRM1, ADβR2) to slow tumor growth and overcome treatment resistance. 

Other medicine-based therapies that have proven to be effective include small molecules that target the trophic signals passed between neurons and tumors. One such signaling molecule that shows potential as a therapeutic target is NGF. Solid cancers leverage NGF signaling as both a source and receiver of this molecule. Breast cancer, prostate cancer, and melanoma cells grow and spread through the dysregulated expression of TrkA and p75^NTR^ receptors, both of which recognize NGF. Binding of these receptors results in downstream PI3K pathway, MAPK pathway, and c-Jun N-terminal kinase (JNK) pathway activation, which drives increased tumor survival, proliferation, and invasion in some cancers [122]. In a leukemia cell line, downregulation of hBex1, a downstream activation target of p75^NTR^, results in resistance to imatinib-induced apoptosis [123]. Although this NGF-related chemoresistance phenotype has not been seen in solid tumors, brain-expressed X-linked (BEX) family alterations are linked to tumorigenesis in lung adenocarcinoma [124] and colorectal cancers [125].

Many solid tumors, including gastric cancer, also release NGF in an effort to influence the surrounding neurons and the TME [57]. In human breast cancer samples, levels of NGF correlate with both the level of tumor innervation and tumor aggression. These data fit a model in which tumor-released NGF stimulates neuron growth and innervation of the tumor. These newborn neurites, in turn, release tumor growth factors, thereby driving tumor aggression. NGF-induced neurons often express tyrosine hydroxylase, suggesting that they deliver catecholamines into the tumor bed [58]. Thus, NGF release from tumors increases the sympathetic innervation of tumors, which, as described previously, drives increased tumor aggression and treatment resistance. 

Given this important role in oncogenic neurotropism, NGF has been identified as a potential target for treatment. Pharmacologic inhibition of Trk receptors showed antitumor effects in preclinical cancer models [126,127,128,129], and current clinical trials are testing the effects of Trk family inhibition in human solid tumors (NCT02097810, NCT02568267, NCT03556228). Additionally, several antibodies against NGF, including tanezumab and fulranumab, have proven to be safe for human use. Anti-NGF antibodies have been shown to be effective in preventing neurite growth into tumors in mice [130] and in mitigating cancer-induced pain in humans [131,132]. However, the use of anti-NGF antibodies in fighting cancer progression in humans has not yet been tested. Future clinical trials testing the antitumor efficacy of anti-NGF therapies will be necessary to understand their role in treating human cancers. 

Many of these medication-based therapies can be taken orally (beta-adrenergic blockers) or intravenously (antibodies). However, the precision of drug targeting can be enhanced in collaboration with surgical techniques. Surgical denervation studies have proven effective in prostate, breast, gastric, and skin cancer mouse models [120,133,134,135], as well as in human patients with gastric cancer [52]. In these studies, the physical separation of the neurons—the source of oncotrophic factors—and the tumors resulted in slowed growth of the tumor, increased sensitivity to chemotherapeutics, and ultimately increased tumor control. Surgical approaches also allow for the direct administration of chemical denervating agents, such as botulinum toxin [136] and small molecule inhibitors of neurotrophic pathways [108]. The effect of localized botulinum toxin delivery to gastric tumors is currently being tested (NCT01822210) and is likely a harbinger of future clinical work in other tumor types. 

## 5. Conclusions

Tumors proliferate and spread throughout the body by taking advantage of neurodevelopmental programs within nearby neural tissues. These programs often respond to neurotrophic proteins, miRNAs, and other trophic signals released by the tumors. The manipulated neurons respond by releasing tumorigenic growth factors in the form of neurotransmitters, and chemokines, which drive tumor growth and spread through both direct and indirect means. These indirect means of stimulating tumor growth are mediated by the TME, which includes stromal, vascular, and immune cells, all of which respond to neurotransmitter and chemokine release from local neurons. Through this tumor-instigated neural-driven manipulation of the TME, tumors increase their access to nutrients and growth factors and evade the immune response, thereby establishing protection from anticancer immune programs. This communication between tumors and neurons is also a vital factor in treatment resistance to exogenous anticancer therapies. 

The neuron–tumor crosstalk described here is often accomplished within the local TME. However, tumors have demonstrated remarkable ability to communicate with distant cellular and neural environments through exosomes and other long-distance signaling methods. Exosomes carry a collection of signaling molecules, including miRNAs, mRNAs, DAMPs, cytokines, chemokines, and other proteins, that are essential for tumor-driven neural manipulation. However, these blood-borne exosomes have also recently been shown to serve as ideal biomarkers. Improved methods of recovering and examining exosome contents now allow for the accurate detection and diagnosis of solid tumors throughout the body. Additionally, these methods improve our understanding of which molecules tumors leverage to manipulate neurons, evade immune recognition, and develop treatment resistance. 

Understanding which molecules are essential for neuron–tumor communication also provides targets for novel therapeutic development. Recent clinical trials support the efficacy of beta-adrenergic blockade in slowing cancer growth and spread. Cholinergic muscarinic blockade has also been found to be effective in human clinical trials. Antibodies and small molecule inhibitors of neurotrophic factors are becoming increasingly recognized as promising anticancer therapies. Additionally, surgical and chemical denervation efforts are proving effective in a variety of cancers. Ongoing and future clinical trials will determine the efficacy of this approach to overcoming treatment resistance in solid tumors, as well as the effect of severing these neurotrophic relationships on tumor growth and spread. We expect these treatments will increasingly be introduced as adjuvant treatments alongside traditional anticancer therapies. 

In tandem with an increased focus on clinical trials that directly test the anticancer effects of targeting neuron–tumor trophic interactions, increased work in model organisms will continue to illuminate the pathways that mediate the observed effects. Controlled genetic and pharmacologic ablation of pathways and signals, combined with advanced transcriptome sequencing, exosome mass spectrometry, and precise tumor phenotyping, will yield discrete steps in the development of tumorigenic neural activity for each cancer type and each tumor context. These experiments will be essential in achieving the goal of improving treatment regimens and improving the lives of patients with solid tumors.

## Figures and Tables

**Table 1:** Active observational clinical trials examining neuron–tumor interactions.

Objective	Tumor of Interest	Trial ID
Document biomarker (protein kinase A) dynamics in cancer tissue	Colorectal cancer	NCT01012804
Determine prognostic value of mu opioid receptor 1 expression/activation in cancer	Colorectal cancer	NCT04353882
Determine prognostic value of SOX2 expression in colorectal cancer	Colorectal cancer	NCT01589900
Assess the ability of common medications * to affect overall survival and disease-free survival	Pancreatic ductal adenocarcinoma	NCT04245644

* Common medications include aspirin (COX1/2 inhibition), statins (HMG-CoA reductase inhibition), metformin, beta blockers, and angiotensin-converting enzyme inhibitors. The mechanism of action for metformin is not entirely understood but includes mitochondrial respiratory chain inhibition, activation of AMP-activated protein kinase, inhibition of glucagon-induced elevation of cAMP, and mitochondrial glycerophosphate dehydrogenase inhibition.

**Table 2:** Active interventional clinical trials targeting neuron–tumor interactions.

Objective	Anticancer Agent	Mechanism of Action	Phase	Tumor Targeted	Route of Administration	Trial ID
Assess the impact of bethanechol therapy on tumor activity	Bethanechol	Nonselective muscarinic activation	1	Pancreatic ductal adenocarcinoma	Oral	NCT03572283
Identify a safe and pharmacologically active dose and regimen for VMD-928 monotherapy	VMD-928	TrkA inhibition	1	Advanced solid tumors or lymphomas not responsive to available therapies *	Oral	NCT03556228
Assess the safety and tolerability of entrectinib therapy	Entrectinib (RXDX-101)	TrkA, TrkB, TrkC, ROS1, ALK inhibition	1	Any locally advanced or metastatic cancer confirmed to be positive for NTRK1, NTRK2, NTRK3, ROS1, or ALK alterations	Oral	NCT02097810
Measure therapeutic response in patients taking entrectinib	Entrectinib (RXDX-101)	TrkA, TrkB, TrkC, ROS1, ALK inhibition	2	Solid tumors that harbor a NTRK1/2/3, ROS1, or ALK gene fusion ^†^	Oral	NCT02568267
Determine the efficacy of carvedilol therapy	Carvedilol	Beta blockade	2	Prostate adenocarcinoma	Oral	NCT02944201
Evaluate ADβR 2/PKA/BAD signal changes following treatment	Propranolol	Beta blockade	2	Prostate carcinoma	Oral	NCT03152786
Evaluate the effects of propranolol and etodolac therapy on recurrence and biomarker expression	Propranolol and etodolac	Beta blockade and COX2 inhibition	2	Pancreatic cancers	Oral	NCT03838029
Obtain the data needed to calculate sample size for a larger controlled trial	Botulinum toxin	Acetylcholine release inhibition	2	Stomach cancer	Injection by gastroscopy	NCT01822210

* These include solid tumors, lymphomas, thymic carcinomas, thymomas, mesotheliomas, head and neck squamous cell carcinomas, ovarian cancers, hepatocellular carcinomas, squamous cell carcinomas of the lung, esophageal cancers, adenoid cystic carcinomas, prostate cancers, cervical cancers, gastric cancers, melanomas, acute myeloid leukemias, pancreatic carcinomas, and non-Hodgkin lymphomas. ^†^ These include breast cancers, cholangiocarcinomas, colorectal cancers, head and neck neoplasms, large-cell lymphomas, anaplastic lymphomas, melanomas, neuroendocrine tumors, non-small cell lung cancers, ovarian cancers, pancreatic cancers, papillary thyroid cancers, primary brain tumors, renal cell carcinomas, sarcomas, salivary gland cancers, and adult solid tumors.
